# Hyperprogression and impact of tumor growth kinetics after PD1/PDL1 inhibition in head and neck squamous cell carcinoma

**DOI:** 10.18632/oncotarget.27563

**Published:** 2020-05-05

**Authors:** Andy Karabajakian, Thibaut Garrivier, Carole Crozes, Nicolas Gadot, Jean-Yves Blay, Frédéric Bérard, Philippe Céruse, Philippe Zrounba, Pierre Saintigny, Charles Mastier, Jérôme Fayette

**Affiliations:** ^1^ Department of Medical Oncology, Centre Léon Bérard, Lyon, France; ^2^ Department of Radiology, Centre Léon Bérard, Lyon, France; ^3^ Department of Pathology, Centre Léon Bérard, Lyon, France; ^4^ Department of Translational Research and Innovation, Centre Léon Bérard, Lyon, France; ^5^ Department of Allergology and Clinical Immunology, CHU Lyon-Sud, Pierre-Bénite, France; ^6^ Department of Head and Neck Surgery, Hôpital Universitaire de la Croix-Rousse, Lyon, France; ^7^ Department of Head and Neck Surgery, Centre Léon Bérard, Lyon, France

**Keywords:** squamous cell carcinoma of head and neck, immunotherapy, programmed cell death 1 receptor, kinetics

## Abstract

**Background:** Hyperprogressive disease (HPD) rate in head and neck squamous cell carcinoma (HNSCC) patients treated with immune checkpoint inhibitors (ICI) was determined using tumor growth kinetics (TGK) and compared with rapidly progressive screen-failure (SF) patients. The impact of TGK on outcomes with salvage chemotherapy (SCT) was also evaluated.

**Results:** HPD was found in 22/120 (18%) patients. Median TGK before the onset of immunotherapy (TGK_pre_) was 2.7 for SF patients and 4.8 for HPD patients, with no significant difference (*p* = 0.17). Disease control rate after initial progressive disease on ICI was 86% with SCT in case of tumor growth deceleration vs 39% in case of tumor growth acceleration.

**Conclusions:** HPD was frequent, but TGK of HPD patients treated with ICI did not differ from SF patients, suggesting that there is no relevant causal relationship between HPD and ICI. After initial PD with ICI, tumor growth deceleration was associated with better outcomes, indicating that TGK_R_ might be useful to detect late responders, meriting prospective investigations.

**Materials and Methods:** TGK ratio (TGK_R_) was defined as the ratio of TGK on ICI (TGK_post_) to TGK_pre_. HPD was defined as TGK_R_ ≥ 2. TGK_R_ >1 indicated tumor growth acceleration, while 0 < TGK_R_ < 1 indicated tumor deceleration.

## INTRODUCTION

Head and neck squamous cell carcinoma (HNSCC) is an aggressive epithelial cancer that derives from mucosa linings of the oral cavity, oropharynx, hypopharynx or larynx. It is also a major public health problem with it being the sixth most incident cancer worldwide, responsible of more than 700 000 cases every year and around 350 000 deaths [1]. Different methods are employed by this aggressive disease to avoid immune recognition, including direct T-cell suppression with soluble or surface inhibitory factors like Programmed-death ligand 1 (PDL1), and the recruitment of immuno-suppressive cell populations [2].

Allison first hypothesized that overcoming the anergic state of T-lymphocytes is possible via the blockade of co-inhibitory signals [3] and research has initially been focused mainly on the PDL1-PDL2/PD1 axis. Immune checkpoint inhibitors (ICI) have since completely revolutionized the treatment of Recurrent/Metastatic (R/M) HNSCC [4–6].

Primary resistance to ICI is frequent across all tumor types, including HNSCC, and concerns almost 60% of patients overall [7]. In a worrying manner, it was suggested that some patients even experience an acceleration of tumor growth kinetics (TGK) on immunotherapy [hyperprogressive disease (HPD)] [8]. Medical charts of 34 patients treated with PD1/PDL1 inhibitors from four different institutions were retrospectively reviewed in 2017 and HPD was found to be frequent (29%) and associated with a worse outcome [8]. Other studies found HPD in different tumor types with varying rates [9, 10] but no consistent predictive genomic or clinical characteristic was associated. All were of a retrospective nature, without a control arm. In consequence, a causal relationship of HPD to ICI has not been proven and a natural evolution of the disease cannot be excluded in the observed cases. Many preclinical studies have hypothesized mechanisms, but no clear biological explanation has seen the day.

Furthermore, after failure of ICI, salvage chemotherapy (SCT) is usually the treatment of choice, but not a lot of data is available on the outcomes in this context. When facing an initial RECIST (Response Evaluation Criteria In Solid Tumors) progressive disease (PD) on ICI, physicians are often confronted with the choice between starting subsequent SCT or continuing ICI in hope for a late response, especially if patients do not experience a worsening general condition. The utility of TGK in this situation is unknown.

Our aim in this study was to know if HPD patients had slower tumor growth before treatment with ICI compared to patients with a naturally exponential growing disease, in which case a strong argument for a causal relationship between ICI and HPD can be made. For this, we used TGK to determine the rate of hyperprogression in clinical trial patients and then compared tumor growth before the onset of immunotherapy with rapidly deteriorating screen-failure (SF) patients. The impact of TGK on outcomes with SCT after initial PD with ICI was also evaluated.

## RESULTS

**Figure 1:**
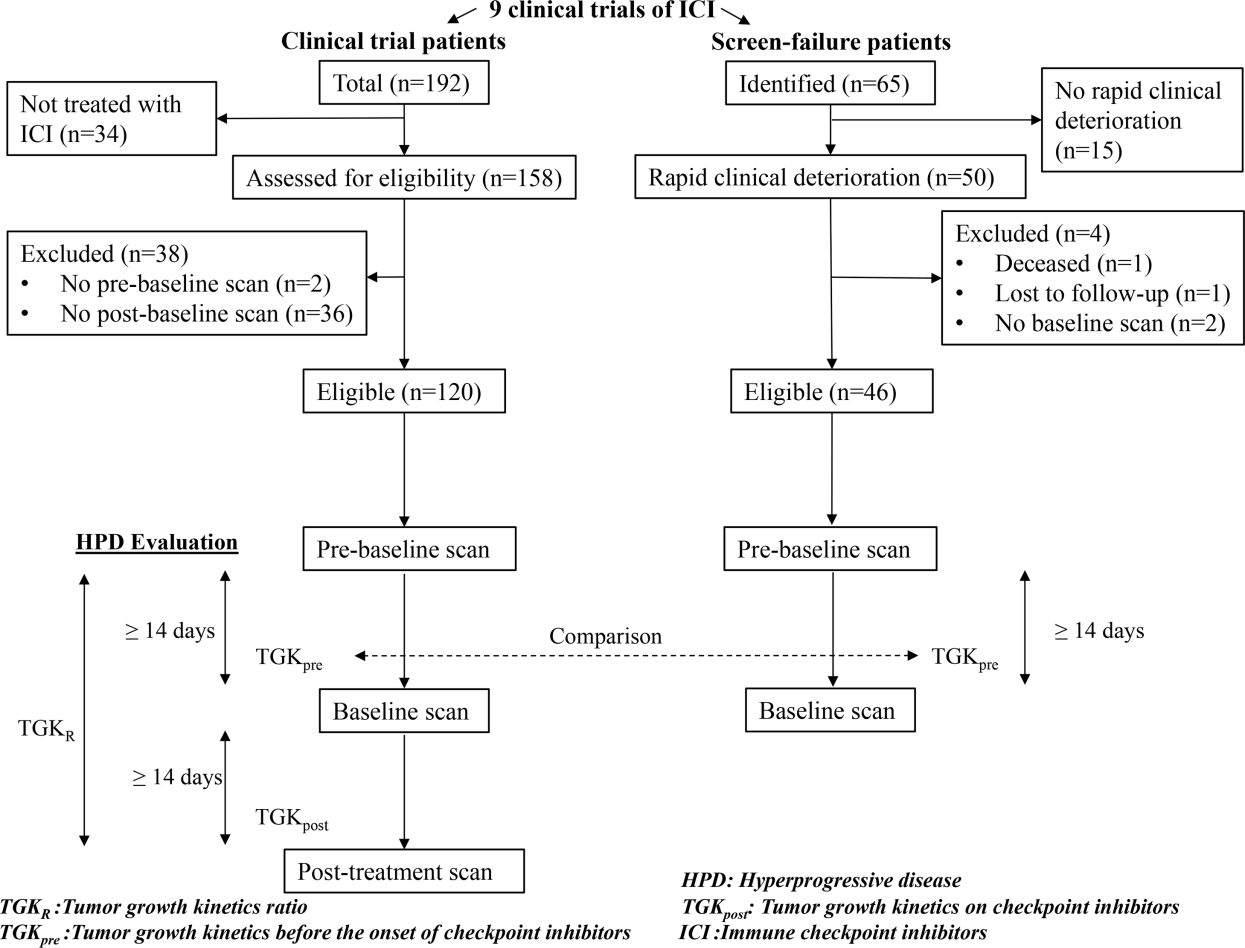
Study flowchart. Hyperprogressive disease (HPD) was evaluated in the clinical trial patients using Tumor Growth Kinetics ratio (TGK_R_). Tumor Growth Kinetics before the onset of immunotherapy (TGK_pre_) was compared with screen-failure patients.

In total, there were 192 patients in 9 clinical trials testing anti-PD1/PDL1 agents alone or in combination with anti-CTLA4 or anti-KIR antibodies. Among those, 158 patients were treated with ICI (the other 34 patients received exclusive chemotherapy) and 120/158 patients were eligible for HPD analysis. The remaining 38 patients were not included in the final analysis because of the absence of available pre-baseline imaging (2 patients) and the absence of post treatment imaging (36 patients) (Figure 1). Median follow-up time since the start of immunotherapy was 34.3 months (95% CI, 32.2 to 35.5).

### Hyperprogressive disease and comparison with screen failure

#### Hyperprogressive disease rate

22/120 (18%) patients had HPD. Median TGK_R_ was 3.2 (95% CI, 2.5 to 4.5). Median TGK_pre_ was 4.8 (95% CI, 1.7 to 7.4) and median TGK_post_ was 17.1 (95% CI, 7.7 to 25).

**Figure 2:**
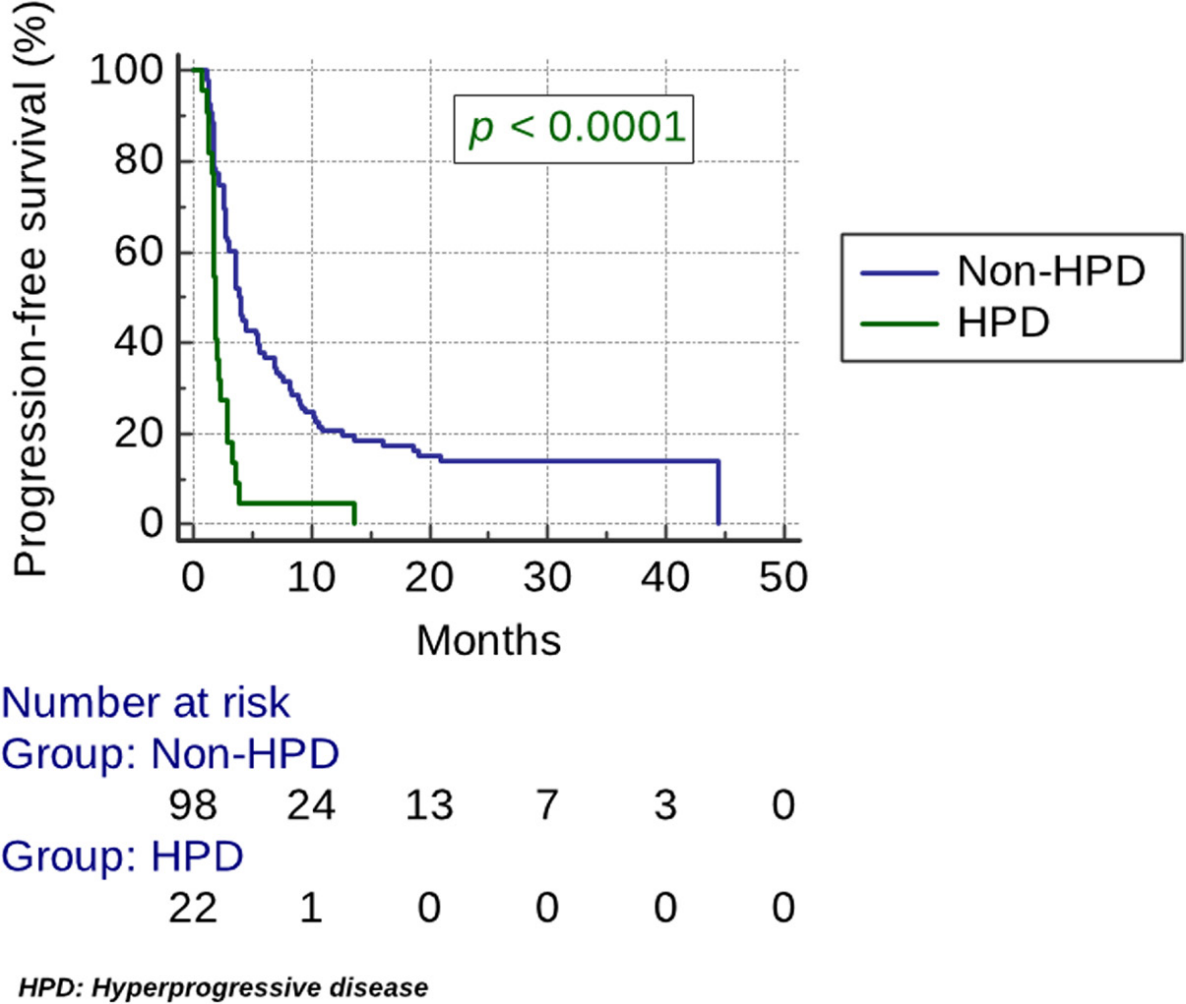
Kaplan–Meier estimates of progression-free survival (PFS). The median PFS was 1.9 months (95% CI, 1.8 to 2.3) in the HPD group vs 3.9 months (95% CI, 3.6 to 5.4). PFS was significantly lower for the HPD group (HR, 2.8; 95% CI, 1.4 to 5.6; *p <* 0.0001).

**Figure 3:**
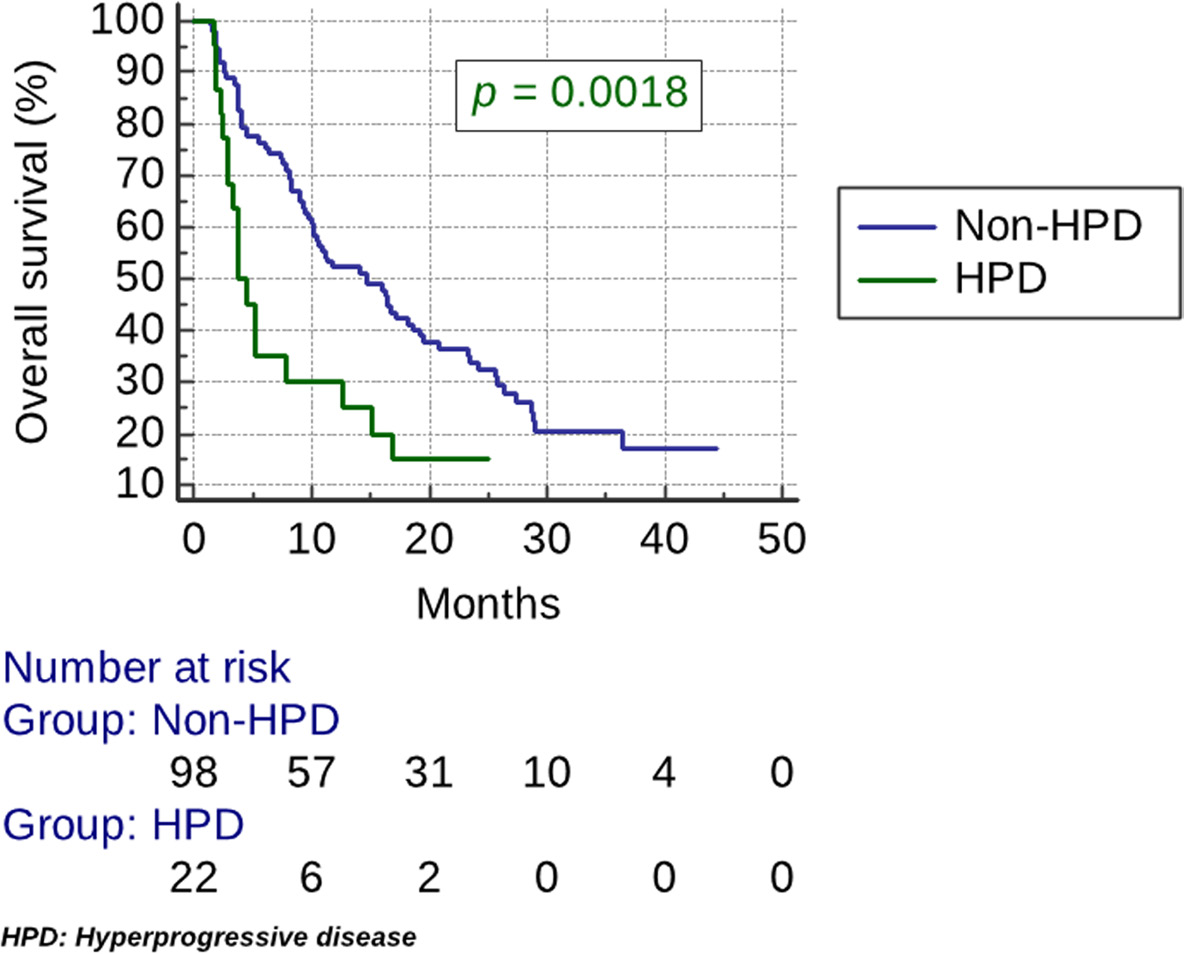
Kaplan–Meier estimates of overall survival (OS). The median OS was 3.8 months (95% CI, 2.8 to 7.8) in the HPD group vs 14.6 months (95% CI, 10.1 to 18.7). OS was significantly lower for the HPD group (HR, 2.2; 95% CI, 1.1 to 4.3; *p =* 0.0018).

HPD was associated with high NLR (*p <* 0.04) (Table 1). No correlation was found with the use of antibiotics, PDL1 or HPV status, elderly age, performance status, disease site, smoking or gender (Table 1). The median PFS was 1.9 months (95% CI, 1.8 to 2.3) in the HPD group vs 3.9 months (95% CI, 3.6 to 5.4). PFS was significantly lower for the HPD group (HR, 2.8; 95% CI, 1.4 to 5.6; *p <* 0.0001) (Figure 2). The median OS was 3.8 months (95% CI, 2.8 to 7.8) in the HPD group vs 14.6 months (95% CI, 10.1 to 18.7). OS was significantly lower for the HPD group (HR, 2.2; 95% CI, 1.1 to 4.3; *p =* 0.0018) (Figure 3).

**Table 1:** Baseline clinical and biological characteristics

Clinical or biological characteristic	HPD (*n* = 22) *N* (%)	Non-HPD (*n* = 98) *N* (%)	*P*
**Neutrophil-to-lymphocyte ratio**			**0.04**
High	13 (59)	48 (48)	
Low/Intermediate	7 (32)	49 (49)	
Unknown	2 (9)	1 (1)	
**Antibiotic use**			0.07
Yes	5 (23)	15 (15)	
No	16 (73)	83 (85)	
Unknown	1 (4)	0 (0)	
**PDL1 status**			0.9
Negative	4 (18)	16 (16)	
Positive	10 (46)	42 (43)	
Unknown	8 (36)	40 (41)	
**Checkpoint inhibitor**			0.62
PD1 based	14 (64)	68 (69)	
PDL1 based	8 (36)	30 (31)	
**Checkpoint inhibition regimen**			1.0
Monotherapy	6 (27)	26 (27)	
Combination	16 (73)	72 (73)	
**Immunotherapy line**			0.16
1^st^ line	7 (22)	48 (49)	
≥ 2^nd^ line	15 (68)	50 (51)	
**Age**			0.35
≥ 65 years	8 (36)	47 (48)	
< 65 years	14 (64)	51 (52)	
**Previous radiation therapy**			0.51
No	4 (18)	13 (13)	
Yes	18 (82)	85 (87)	
**Human papillomavirus**			0.82
Negative	12 (55)	46 (47)	
Positive	2 (9)	10 (10)	
Unknown	8 (36)	42 (43)	
**Performance status**			0.45
0	4 (18)	31 (32)	
1	17 (77)	64 (65)	
≥ 2	1 (5)	3 (3)	
**Gender**			1.0
Male	18 (82)	79 (81)	
Female	4 (18)	19 (19)	
**Smoking status**			0.75
Non-smoker	4 (18)	15 (15)	
Previous/current smoker	18 (82)	83 (85)	

Abbreviation: HPD, hyperprogressive disease.

#### Hyperprogressive disease rate with total tumor burden

When calculating TGK_R_ with TTB, HPD was found in 21/120 (17.5%) patients. Median TGK_R_ was 3.2 (95% CI, 2.4 to 4.7). HPD was concordant between RECIST 1.1 and total tumor burden evaluation for 16/22 (73%) patients.

#### SF tumor growth kinetics comparison

In total, 65 patients were screen-failed in the 9 clinical trials. Of these, 50 SF cases were attributed to rapid clinical deterioration and were included in the final analysis (Figure 1). The following reasons were the cause of SF in the included patients: death, symptomatic cerebral metastases, elevated liver enzymes attributed to metastatic disease, corticosteroid use for disease control and worsening general condition. 46/50 patients were eligible for TGK_pre_ assessment as 1 patient was deceased, 1 patient was lost to follow up and 2 patients didn’t have an available CT-scan.

**Figure 4:**
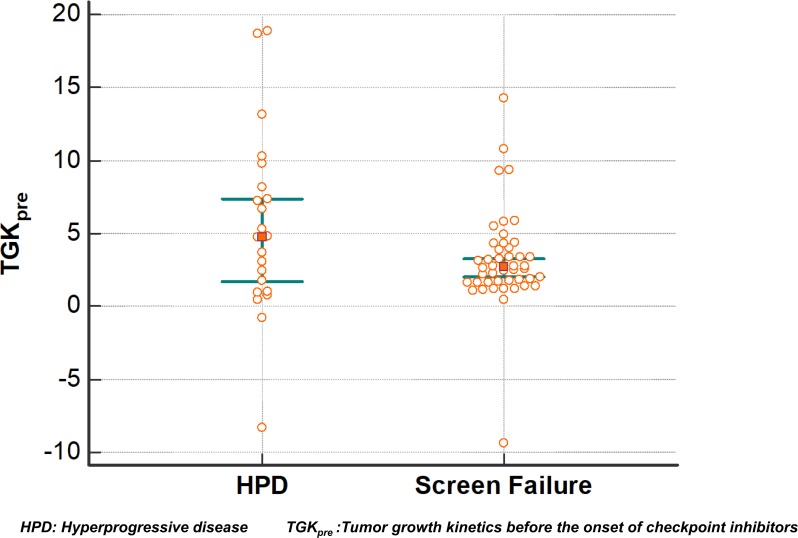
Tumor growth kinetics before the onset of immunotherapy (TGKpre). Each dot represents a distinct TGK_pre_ value. Overlapping confidence intervals of this dot plot show that distribution is similar.

Median TGK_pre_ was 2.7 (95% CI, 2 to 3.3). No significant difference in TGK_pre_ with HPD patients was found using a Mann–Whitney test (*p =* 0.17) (Figure 4).

### Tumor growth kinetics and salvage chemotherapy

#### Outcomes on salvage chemotherapy

Out of 158 patients treated with ICI, 67 patients were eligible. ICI were given as monotherapy in 31% of patients or as combination in 69%. Salvage chemotherapy included platinum-based regimen (55%), taxane-based regimen (21%), capecitabine (3%), cetuximab (8%), vinorelbine (1%) and methotrexate (12%). Cetuximab was administered in combination with platinum or taxanes in 14% of patients. The median number of prior treatment lines was 2 (range 1–5). The ORR (Objective response rate) was 28%. 6 patients (9%) presented CR (4 with platinum-based chemotherapy, 1 with Docetaxel and 1 with cetuximab) and 13 patients (19%) had PR. The DCR was 61%. The median PFS was 3.5 months (95% CI, 2.5 to 4.9) and the median OS was 9 months (95% CI, 7.2 to 13.8).

#### TGKR after initial progression on checkpoint inhibitors

**Figure 5:**
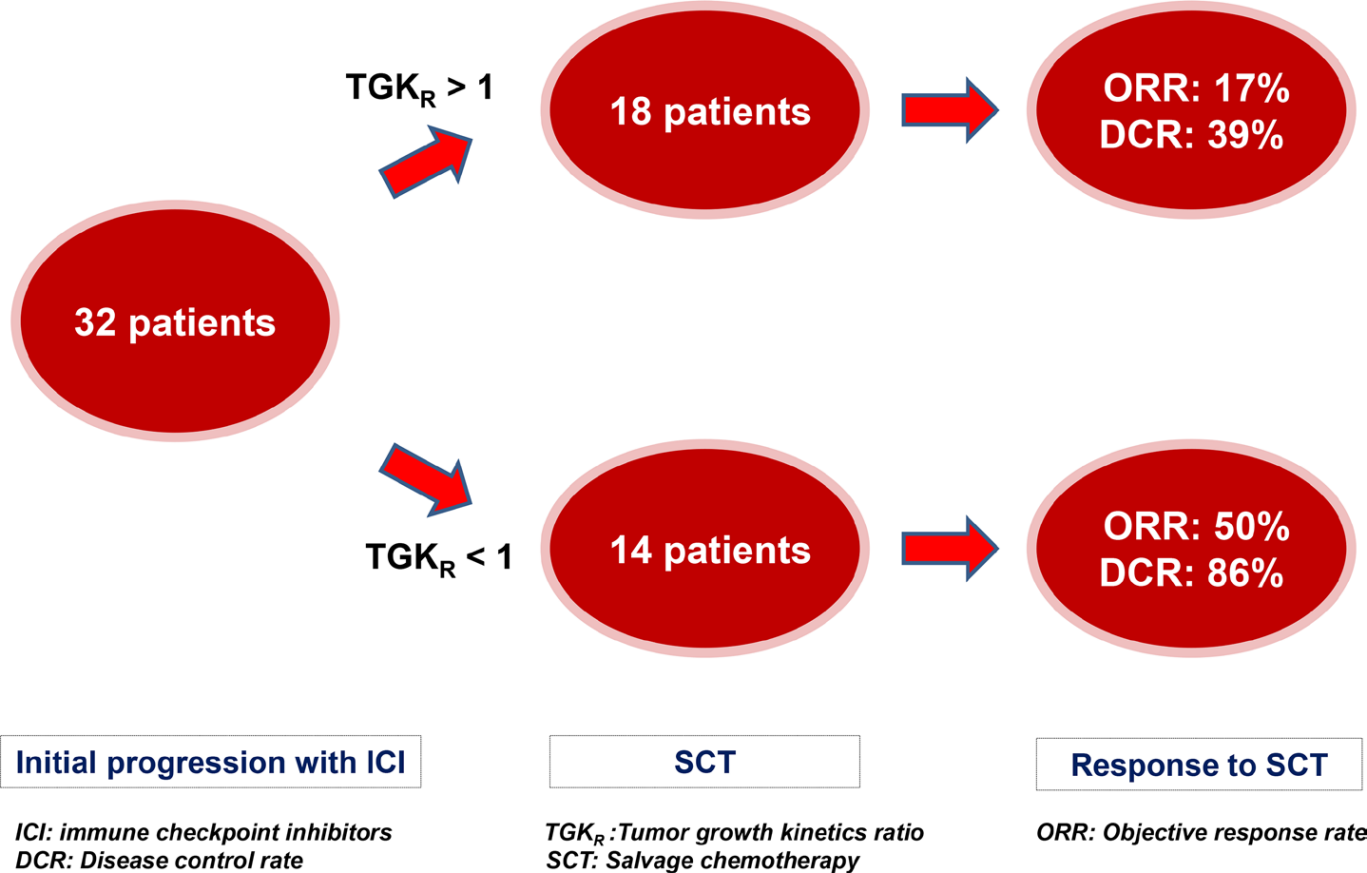
Impact of Tumor Growth Kinetics ratio (TGKR) on outcomes with salvage chemotherapy (SCT) after initial RECIST 1.1 progression with immune checkpoint inhibitors (ICI). Patients were more likely to respond to SCT in case of tumor growth deceleration (0 < TGK_R_ < 1) and had higher disease control rate (DCR) than those with tumor growth acceleration (TGK_R_ > 1).

Out of 39 patients who presented initial RECIST 1.1 PD with ICI and were subsequently treated with salvage chemotherapy, 32 patients were eligible for TGK_R_ assessment. 7 patients were ineligible because of the absence of pre-baseline scan. Seven (7) out of 14 (50%) patients with disease deceleration (TGK_R_ < 1) on ICI had PR or CR and 5 (36%) had SD. DCR was 86%. 3 out of 18 (17%) patients with disease acceleration (TGK_R_ > 1) on ICI had PR and 4 (22%) had SD. DCR was 39% (Figure 5). Median time from last ICI administration to first imaging on salvage chemotherapy was 2.3 months (95% CI, 2 to 2.7).

## DISCUSSION

### Hyperprogression: a real phenomenon?

TGK did not differ between rapidly deteriorating SF and ICI-treated HPD patients, strongly suggesting that there is no relevant causal relationship between HPD and ICI, at least in some cases. In other words, patients that are considered to have ICI-induced hyperprogression have a tumor growth before ICI treatment that is similar to patients that have a rapidly evolving disease, not treated with ICI. Exponentially growing tumors are expected to continue to grow exponentially when untreated.

In our study, we used TGK to evaluate hyperprogression as this was the methodology used in the first published work on HPD in HNSCC by *Saâda-Bouzid et al* [8]. If another definition was used [e.g., 50% Tumor growth ratio (TGR) in 4 weeks], results might have been different, highlighting the need of an international consensus to define hyperprogression. More recently, HPD evaluation in non-small-cell lung cancer (NSCLC) patients was highly concordant using TGK and TGR which suggests the interchangeability of these two methods [11].

Multiple mechanisms have been raised: oncogenic signaling activation [11], upregulation of alternative immune checkpoints [12], major immune reactions caused by PD1/PDL1 inhibitors, previous irradiation [8], tumor proliferation via a direct (DNA damage with free radicals) or indirect (angiogenesis and tissue remodeling promotion) effect [13–15], expansion of PD1-expressing T-regulatory cells and modulation of tumour-promoting cells [16]. Interaction with tumor-associated macrophages via the Fc domain of the anti-PD1 antibody with reprogramming into immunosuppressive M2-like macrophages has also been incriminated [17]. No consistent predictors have yet been validated, further supporting our results.

On the other hand, 36 patients in our cohort were excluded because of the absence of post-baseline imaging that might be due to in some cases to hyperprogression-related death. This may lead to underestimating the phenomenon, although some of these deaths might also be due to disease natural behavior.

The absence of randomization and the difference in screening windows could of course be a source of bias, but we believe that our comparison with rapidly deteriorating SF patients is pertinent as those are the patients that might best represent a naturally exponential growing disease.

Furthermore, the heterogeneity of the immunotherapy regimens in this study could also be a confounding factor, but a previous work evaluating HPD in early-phase immunotherapy trials found hyperprogression with combination ICI as well as monotherapy, with no significant difference [18], mirroring our results.

We chose to compare growth kinetics before starting immunotherapy (TGK_pre_) between the two groups (and not the ratio of TGK_post_ to TGK_pre_), as we thought that this was the most appropriate time interval to show similarity in the speed of disease progression. SF patients were often lost to follow-up, received ICI in another setting or had variable timing in CT-scan evaluation after screen-failure, so comparison of the ratio of TGK_post_ to TGK_pre_ would have been less appropriate.

In addition, we have found that HPD evaluation with total tumor burden and with RECIST 1.1 was concordant for about seventy percent of patients only, further emphasizing the need to find a consensus in HPD diagnostic criteria.

This is the largest cohort evaluating HPD in HNSCC to date. Our findings underscore the difficulties in interpreting the evolution of the disease under ICI and present an important message to clinicians that are treating patients with R/M HNSCC.

We found that hyperprogression was frequent (18%) and correlated only with a high NLR. Previous retrospective studies (albeit with less included patients) have already shown a correlation of high NLR with poor outcomes [19, 20], and so our results confirm this literature data, even if the relative small number of HPD patients might have weakened the statistical power of the comparison.

### Impact of tumor growth kinetics

Some chemotherapeutic agents have been shown to exert immune-reactive events such as upregulation of MHC (major histocompatibility complex) class molecules and increased antigen presentation [21, 22]. Chemotherapy can augment tumor immunity by decreasing the number of Immunosuppressive cells like myeloid-derived suppressor cells (MDSC) or T-regulator cells in the microenvironment which can lead to an accumulation of helper T-cells on site [23, 24] and by promoting anti-tumor CD4+ T-cell phenotype [25]. In fact, Improved responses to chemotherapy has been reported after vaccination immunotherapy in various tumor types [26].

Our results confirm that salvage chemotherapy seems to be more effective with better outcomes (ORR, PFS, and OS), suggesting that chemotherapy is enhanced by immunotherapy. Indeed, ORR was 28%; which is higher than the historical controls before the era of immunotherapy; varying between 6 and 24% [27–29].

By showing that patients who presented initial PD on ICI associated to a tumor deceleration (TGK_R_ < 1) had higher ORR that those with tumor acceleration (TGK_R_ > 1), we think that some of the responses in our study were due/enhanced by circulating anti-PD1/PDL1 antibodies causing delayed onset of response. The median time from the last immunotherapy administration and the first evaluation on salvage chemotherapy was 2.3 months, which is compatible with the half-life of these agents [30, 31]. We by consequence also believe that TGK_R_ might be a useful tool to help select potential late responders to ICI and thus avoid toxic and possibly inefficient chemotherapy.

Of course, this hypothesis should be evaluated in other data sets and if confirmed, it could potentially be tested prospectively by randomizing to continued immunotherapy versus switch to salvage chemotherapy in case of tumor growth decelaration.

In conclusion, HPD was found in 18% of cases and correlated with high NLR. Growth kinetics before ICI-treated HPD patients were similar to SF patients, suggesting that the published rates of HPD might be due to natural disease behavior, at least in some cases. After initial RECIST PD with ICI, tumor growth deceleration was associated with better outcomes compared to tumor growth acceleration, indicating that TGK_R_ might be useful to detect late responders and avoid SCT, meriting prospective investigations.

## MATERIALS AND METHODS

### Patient selection and data collection

All patients with a histologically confirmed R/M HNSCC treated in Léon Bérard cancer center in a clinical trial testing PD1/PDL1 antibodies alone or in combination with an anti-Cytotoxic T-Lymphocyte Antigen 4 (CTLA4) or an anti-Killer Immunoglobulin-like Receptor (KIR) antibody between March 2014 and November 2018; were included (Figure 1). All imaging was retrospectively reviewed by medical oncologists and independent radiologists. SCT was defined as the first line of chemotherapy administered after failure of ICI. The following data was collected and recorded: age, gender, primary tumor location, tobacco use, human papilloma virus (HPV) status (p16 immunostaining and/or DNA *in situ* hybridization), previous multimodal therapy at the initial stage, neutrophil-to-lymphocyte ratio (NLR) (0 to 72 hours before start of treatment; with a cut-off ≥5 defining high NLR), antibiotics intake in the 3 months prior to immunotherapy, the number and dates of previous and following lines of systemic therapy. Also collected was the best overall response on and after failure of immunotherapy using RECIST 1.1, progression-free survival (PFS) and overall survival (OS).

PD-L1 expression in archival formalin-fixed, paraffin-embedded (FFPE) samples was assessed. Samples were provided by the local Biological Resources Center (BB-0033-00050, CRB Centre Léon Bérard, Lyon France). 4-μm thick tissue sections of FFPE tissue were prepared according to conventional procedures. For each sample, hematoxylin and eosin (HES) staining was performed to determine the number of tumor cells. Immunohistochemistry (IHC) was performed on an automated immunostainer (Ventana Benchmark ultra, Roche, Meylan, France) using Ultra View DAB Kit according to the manufacturer’s instructions. Sections were incubated with a rabbit monoclonal human anti-PDL1 Ab (diluted at 1:50, Quartett, Berlin, Germany) clone QR1. The Ventana amplification kit was used and an anti-rabbit-HRP was applied on sections. Staining was visualized with DAB solution with 3,3′-diaminobenzidine as a chromogenic substrate. Finally, the sections were counterstained with Gill’s hematoxylin. All samples were examined by a qualified anatomopathologist for combined positive score (CPS), defined as the number of PD-L1-positive cells (tumour cells, macrophages and lymphocytes) divided by the number of tumour cells × 100 (a minimum of 100 viable tumour cells must have been present for the specimen to be considered evaluable). CPS ≥ 1 was the cut-off for PDL1 positivity.

The data collection cutoff point was December 4, 2019.

### Hyperprogressive disease definition

In order to be eligible for TGK_R_ assessment, patients had to have a pre-baseline scan, a baseline scan and post-treatment scan. Minimal delay between 2 CT-scans was 14 days and patients had to have started ICI therapy in the 6 weeks following baseline scan (Figure 1). TGK before (TGK_pre_) and after (TGK_post_) anti-PD1/PDL1 therapy were evaluated. TGK_pre_ was defined as the difference of the sum of the largest diameters of the target lesions per unit of time between pre-baseline and baseline imaging [(S_0-_S_pre_)/(T_0_-T_pre_)]. TGK_post_ was defined in the same manner between on immunotherapy and baseline imaging [(S_POST_-S_0_)/(T_POST_-T_0_)]. HPD was defined as TGK_R_ (ratio of TGK_post_ to TGK_pre_) ≥ 2. TGK_R_ > 1 indicated tumor growth acceleration, while 0 < TGK_R_ < 1 indicated tumor deceleration. TGK_R_ < 0 indicated tumor shrinkage.

TGK_R_ was calculated with RECIST 1.1 for all patients. Since TGK only evaluates the variation of target lesions and does not include new lesions in the assessment of tumor growth, TGK_R_ was also calculated using total tumor burden (TTB).

### Screen-failure inclusion

All patients that were screened for the same clinical trials and were subsequently ineligible were analyzed. Patients that were considered screen-failed because of rapid clinical deterioration attributed to disease progression were included in the ‘screen failure’ (SF) group. Patients were not included if they were subsequently treated with ICI in another clinical trial or setting. TGK_pre_ was calculated in this group in the same manner in order to compare tumor growth with HPD patients before the onset of immunotherapy (Figure 1).

### Statistical analysis

Descriptive statistics were used to summarize the baseline characteristics of the patients. Chi-squared or Fisher’s exact test was used for statistical comparisons of categorical data. Mann–Whitney test was used to compare distribution. Partial response (PR) and complete response (CR) defined objective response (OR). Disease control rate (DCR) was defined as the sum of CR, PR, and stable disease (SD). PFS time was defined as the period from the date of initial treatment administration to the date of clinical disease progression, mortality from any cause or the last follow-up. OS time was defined as the period from the date of initial treatment administration to the date of mortality from any cause or the last follow-up. The Kaplan–Meier method was used to assess PFS and OS. Comparison was done using the log-rank test. Data of patients who were lost to follow-up were censored at the time of last contact. Statistical analysis was done using MedCalc 18.11.6 statistical software (MedCalc Software, Mariakerke, Belgium).

## Abbreviations

HNSCC: Head and neck squamous cell carcinoma
PDL1: Programmed-death ligand 1
ICI: Immune checkpoint inhibitors
R/M: Recurrent/metastatic
TGK: Tumor growth kinetics
HPD: Hyperprogressive disease
SCT: Salvage chemotherapy
RECIST: Response Evaluation Criteria In Solid Tumors
PD: Progressive disease
SF: Screen-failure
TGR: Tumor growth ratio
NSCLC: Non-small-cell lung cancer
MHC: Major histocompatibility complex
MDSC: Myeloid derived suppressor cells
CTLA4: Cytotoxic T-Lymphocyte Antigen 4
KIR: Killer Immunoglobulin-like Receptor
HPV: Human papilloma virus
NLR: Neutrophil-to-lymphocyte ratio
PFS: Progression-free survival
OS: Overall survival
FFPE: Formalin-fixed, paraffin-embedded
HES: Hematoxylin and eosin
IHC: Immunohistochemistry
CPS: Combined positive score
TGK_pre_: TGK before anti-PD1/PDL1 therapy
TGK_post_: TGK after anti-PD1/PDL1 therapy
TTB: Total tumor burden
PR: Partial response
CR: Complete response
OR: Objective response
DCR: Disease control rate
SD: Stable disease
