# Addendum: Hyperprogression and impact of tumor growth kinetics after PD1/PDL1 inhibition in head and neck squamous cell carcinoma

**DOI:** 10.18632/oncotarget.28316

**Published:** 2022-12-06

**Authors:** Andy Karabajakian, Thibaut Garrivier, Carole Crozes, Nicolas Gadot, Jean-Yves Blay, Frédéric Bérard, Philippe Céruse, Philippe Zrounba, Pierre Saintigny, Charles Mastier, Jérôme Fayette

**Affiliations:** ^1^ Department of Medical Oncology, Centre Léon Bérard, Lyon, France; ^2^ Department of Radiology, Centre Léon Bérard, Lyon, France; ^3^ Department of Pathology, Centre Léon Bérard, Lyon, France; ^4^ Department of Translational Research and Innovation, Centre Léon Bérard, Lyon, France; ^5^ Department of Allergology and Clinical Immunology, CHU Lyon-Sud, Pierre-Bénite, France; ^6^ Department of Head and Neck Surgery, Hôpital Universitaire de la Croix-Rousse, Lyon, France; ^7^ Department of Head and Neck Surgery, Centre Léon Bérard, Lyon, France

**This article has an addendum:** Patient consent was obtained from the patient. This study was approved by the ethical review board of Centre Léon-Bérard.

Original article: Oncotarget. 2020; 11:1618–1628. 1618-1628. https://doi.org/10.18632/oncotarget.27563

